# Incremental diagnostic value of mycobacterium tuberculosis antigen-based skin test for active tuberculosis: a diagnostic accuracy study

**DOI:** 10.3389/fimmu.2026.1787918

**Published:** 2026-05-26

**Authors:** Fan Jiang, Ya Yu, Xianghua Kong, Linyang Li, Ping Han, Jing Dong, Zhujun Li, Rui Yu, Bo Wu

**Affiliations:** 1 Fifth Department of Tuberculosis, Chongqing Public Health Medical Center, Chongqing, China; 2 Department of Tuberculosis Prevention and Control, Chongqing Municipal Institute of Tuberculosis, Chongqing, China

**Keywords:** C-TST, diagnosis, TBST, tuberculin skin test, tuberculosis

## Abstract

**Background:**

In WHO consolidated guidelines on tuberculosis (TB) for TB infection tests, studies to assess the diagnostic association for active TB compared with current infection tests were recommended as one of the priorities for future research. Previous research has indicated some clinical characteristics of Creation Tuberculin Skin Test (C-TST) which were different from Tuberculin Skin Tests (TST). Based on these new clinical characteristics, the research objective was to evaluate the incremental diagnostic value of C-TST for the diagnosis of active TB under specific conditions, compared with TST.

**Methods:**

This was a retrospective, non-randomized, exploratory diagnostic accuracy study. The incremental diagnostic value of C-TST above a certain cut-off point was evaluated in suspected Pulmonary Tuberculosis (PTB) cases, and was compared with TST, which was conducted in Chongqing between January 2022 and July 2024.

**Results:**

In 1553 suspected TB participants, 398 were tested using TST or C-TST. In active PTB cases, the median diameter of C-TST induration or erythema was significantly larger than that of TST induration (*W*=5173.5, *P*<0.001). When using the third quartile of C-TST with active TB (34.3 millimeter) as the cut-off point, positive predictive value (PPV) of C-TST for active TB diagnosis was 74.5% (95%CI, 63.8%-85%) which was significantly larger than that of TST (*Z*=2.46, *P=0.014*).

**Conclusions:**

At a specific cut-off value, C-TST shows preliminary exploratory potential for indicating active TB among adult suspected PTB cases. Further external and prospective validation is still warranted to confirm its clinical diagnostic utility.

## 1 Introduction

Tuberculosis (TB) has returned to being the world’s leading cause of death from a single infectious agent, consistently appearing among the top 10 causes of death worldwide (1). Globally, the total estimated number of incident cases was 10.7 million, and the reported number of notified cases was 8.3 million, causing a global gap of 2.4 million. The net reduction of TB incidence rate between 2015 and 2024 was 12%, which fell far short of World Health Organization (WHO) End TB Strategy milestone of a 50% reduction by 2025 (1). Ending the global TB epidemic remains a distant goal.

One of the keys to controlling TB epidemic is rapid and accurate diagnostic testing for active TB and TB infection (2, 3). The current laboratory diagnosis of TB is still primarily based on microbiological detection of *Mycobacterium tuberculosis* (Mtb). But of the 6.9 million people diagnosed with pulmonary TB (PTB) worldwide, only 64% were bacteriologically confirmed in 2024 (1).

The diagnosis of the bacteriologically negative TB relies on symptom screen, imaging evidence, and tests for TB infection like tuberculin skin test (TST) and interferon-gamma release assay (IGRA) (3, 4). These methods usually lack specificity in the diagnosis of active TB, which can lead to missed diagnosis, indicating an urgent need to develop new tools with better diagnostic association. In WHO consolidated guidelines on tuberculosis for TB infection tests, longitudinal studies to assess the diagnostic association for active TB compared with current infection tests were recommended as one of the priorities for future research (3).

In 2022, WHO issued recommendations on the use of Mycobacterium tuberculosis antigen-based skin test (TBST) which may be used to test for TB infection, including Cy-Tb (Serum Institute of India, India), Diaskintest (Generium, Russian Federation); and C-TST (Anhui Zhifei Longcom, China). TB infection diagnostic performance of TBST compared with TST or IGRA has been systematically assessed, which appeared to perform similarly to IGRA or TST (5). However, there were currently few studies applying TBST in suspected TB cases to predict active TB.

Since the C-TST was approved for the diagnosis of TB infection in China in 2020, considerable data and experience has been accumulated in high-risk groups such as adolescents and suspected TB cases (6). Some clinical characteristics of C-TST have been discovered, which were different from TST. Based on these new clinical characteristics, C-TST may have the ability to predict active TB by identify recent TB infection under specific conditions. This possibility has the potential to clarify the incremental diagnostic value of C-TST for the diagnosis of active TB, and needs further investigation.

## 2 Methods

### 2.1 Study design

This was a retrospective diagnostic accuracy study comparing C-TST and TST under specific conditions, which was conducted among suspected PTB cases in Chongqing Municipal Institute of Tuberculosis between January 2022 and July 2024 by Chongqing Municipal Institute of Tuberculosis. This study was reported according to STARD 2015 (7).

In this study, the diagnostic research question was reflected by “PIRO”. “P” represented participants who were adult suspected PTB cases in Chongqing, “I” represented index tests which were C-TST and TST, “R” represented China national diagnostic criteria for PTB, and “O” represented outcomes including sensitivity, specificity, positive predictive value (PPV), negative predictive value (NPV), and receiver operating characteristic (ROC) curve (8). We evaluated the C-TST diagnostic accuracy above a certain cut-off point in suspected PTB cases, and compared it with TST in Chongqing, China. This cut-off selection and diagnostic performance analysis should be regarded as exploratory rather than confirmatory.

Adult suspected PTB cases were defined as those with abnormal chest imaging findings consistent with the manifestations of PTB, including primary and secondary PTB, hematogenous disseminated PTB, bronchial TB, and TB pleurisy (9). According to the health policy of China, suspected PTB cases should be screened for active PTB in designated medical institutions (10).

The participants were diagnosed according to China national diagnostic criteria for PTB (9). The diagnosis of PTB was primarily based on etiological examination including bacteriology and molecular biology, combined with epidemiological history, clinical manifestations, chest imaging, relevant auxiliary examinations, and differential diagnosis, to make a comprehensive analysis and diagnosis. Etiological and pathological results were used as the basis for confirmation. Immunological test were the most important part of auxiliary examination, including TST and IGRA.

TST remains the most widely used tool at a global scale. According to China national diagnostic criteria for PTB (9), the TST induration cut-off point of ≥10mm was used to indicate TB infection in diagnosis of PTB considering that Bacillus Calmette-Guérin (BCG) vaccination at birth has been a national policy in China, and coverage is at a very high level, which might interfere with TST reactivity.

C-TST is a type of TBST, which uses a new type of recombinant fusion protein that only contains Mycobacterium TB early secretory antigen target six (ESAT-6) and culture filtrate protein ten (CFP-10), and exposure to previous BCG vaccine will not interfere with the C-TST reactivity. The C-TST induration or erythema cut-off point of ≥5mm has been commonly recommended for TB infection. C-TST has not been included in the current China national diagnostic criteria for PTB.

### 2.2 Sample size

According to the previous diagnosis investigation in suspected PTB cases by Chongqing Public Health Medical Center and Chongqing Municipal Institute of Tuberculosis, the C-TST PPV was about 70% when the cut-off value was above the median of the test results, and the TST PPV was about 50%. The sample size was estimated at 73 suspected PTB cases in each group using the following formula, based on the allowable error of 20%, and the significance level of 5%. After applying the Yates’ continuity correction, the sample size in each group was 83 (11).

α represents the significance level, and β represents the allowable error in the following formula. *P_1_* represents C-TST PPV when the cut-off value is above the median of the test results in the C-TST group, and *P_2_* represents TST PPV when the cut-off value is above the median of the test results in the TST group.


$$N=\frac{{\left[Z_{\alpha }\sqrt{2\times \bar{P}(1-\bar{P})}+Z_{\beta }\sqrt{P_{1}(1-P_{1})+P_{2}(1-P_{2})}\right]}^{2}}{{\left(P_{1}-P_{2}\right)}^{2}}$$

### 2.3 Study participants

The inclusion criteria were as follows: (1) participants at least 18 years old, (2) participants who met the definition of suspected PTB cases according to the national diagnostic criteria for PTB, (3) participants who underwent IGRA, and (4) participants who can participate in the entire PTB diagnosis process. The exclusion criteria were as follows: (1) participants without PTB manifestations on chest imaging, (2) participants with C-TST or TST results that were not properly recorded which were required to record the transverse diameter and longitudinal diameter, (3) participants with HIV positivity.

Participants with comorbidities were included in this real-world exploratory study to reflect actual clinical practice. According to the inclusion and exclusion criteria, participants were continuously selected until they were fully enrolled in Chongqing Municipal Institute of Tuberculosis which was a designated medical institution for PTB.

### 2.4 Randomization and confounding factors

Since this study was retrospective, it was no longer possible to assign suspected PTB cases to the C-TST group and the TST group using randomization methods. The actual use of TST and C-TST was mainly determined by the convenience of the result read time and the physicians’ preference, with all participants being enrolled consecutively.

Enrollment of participants did not take into account different levels of disease severity, different stages of disease progression, different symptoms and signs, whether treated, and presence or absence of complications to avoid spectrum bias.

The C-TST or TST results were read by the physicians who were aware of the clinical information of the participants, so the read of skin tests results could not be blinded to the diagnosis of PTB completely because the etiological and other test results may precede the read of the skin test.

### 2.5 Procedures

Standardized training was provided for all medical workers involved in this study, including clinical theory, as well as TST and C-TST practical skill. The trainees were assessed, and only those who passed the assessment could implement PTB diagnosis.

C-TST and TST were performed according to the Mantoux technique. When performing TST (Purified Protein Derivative of Tuberculin; Xiang Rui, China or Chengdu Institute of Biology, China), 0.1 mL (5 IU) purified protein derivative of tuberculin was injected on the volar surface of the left forearm intradermally. The induration was measured in millimeter after 72 hours at injection sites.

When performing C-TST (Recombinant Mycobacterium Tuberculosis Fusion Protein; Zhifei Longcom Biologic Pharmacy Company, Anhui, China), 0.1 mL (5U) recombinant mycobacterium TB fusion protein was injected on the volar surface of the left forearm intradermally. The induration or erythema was measured in millimeter after 48 hours at injection sites.

The diagnosis of all participants has been confirmed through follow-up, with a period of 2 months. In this study, there were no indeterminate results in the diagnosis.

For each enrolled participant, socio-demographic information and diagnostic information were collected from hospital information system (HIS), including age, gender, consultation time, medical history, PTB related symptoms, imaging findings, diagnostic results, and C-TST/TST reading results et al.

### 2.6 Statistical analysis

We calculated sensitivity, specificity, PPV, and NPV, as well as their 95% confidence intervals (CI). ROC curve has been plotted comparing C-TST and TST diagnostic accuracy. The diagnostic efficacies of different ROC curves were compared using the Delong test.

Different rates were tested using chi-square test. Comparison of different mean diameters used the t-test for those that conform to the normal distribution, and comparison of different median diameters used the Mann-Whitney U test for those that do not conform to the normal distribution. The Shapiro-Wilk test was used to determine whether the data followed a normal distribution.

Some participants were unable to verify the C-TST or TST results due to leave or other reasons, resulting in missing data. Missing data were excluded from the analysis when they constituted less than 5% of the dataset and the missingness was completely at random.

A two-sided P<0.05 was taken as statistically significant. Statistical analyses were performed using R Statistical Software (v4.4.2; R Core Team 2024). All statistical graphs were also created using R Statistical Software (v4.4.2; R Core Team 2024).

## 3 Results

### 3.1 The screening process and baseline data for participants

**Figure 1:**
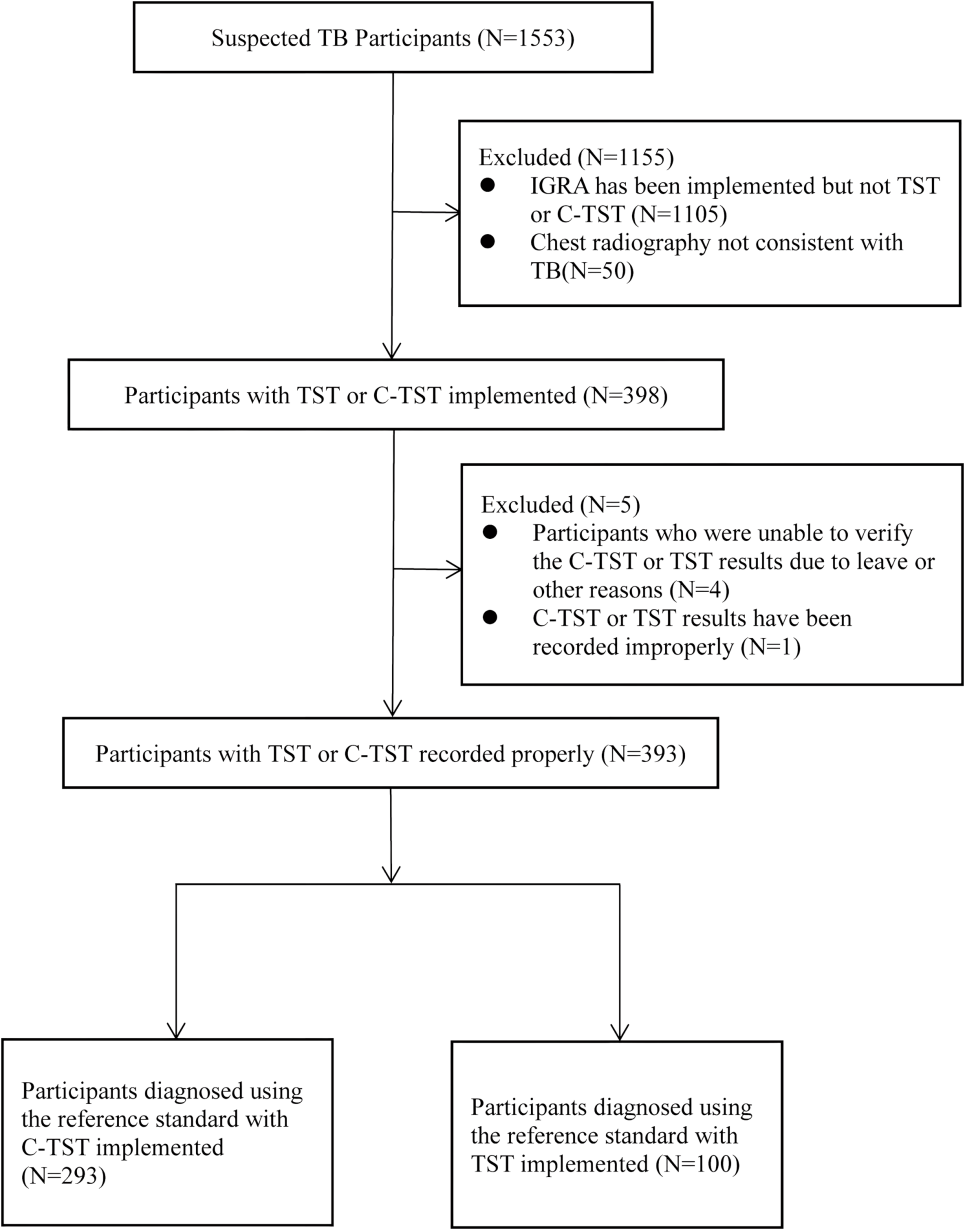
Flow chart of suspected TB participants screened for active TB.

There were 1553 suspected TB participants in Chongqing Municipal Institute of Tuberculosis between January 2022 and July 2024, and 1155 (74.4%) were excluded for various reasons, leaving 393 (25.3%) participants with TST or C-TST result (**Figure 1**).

There were 293 suspected TB participants diagnosed using the reference standard with C-TST implemented, and 100 suspected TB participants with TST implemented. Participants who were unable to verify the TST or C-TST results due to leave or other reasons were excluded from statistical analysis, and participants whose results of TST or C-TST were recorded improperly have been excluded too. This situation has caused missing data, which were completely random with a low proportion of 1.3% (5/398) (**Table 1**).

**Table 1:** Suspected TB participants screened for active TB between January 2022 and July 2024 in Chongqing.

Characteristics	Subgroup	Participants with C-TST result	Participants without C-TST result	Participants with TST result	Participants without TST result
Gender	Male	174	1	61	1
	Female	119	1	39	2
Age	18-34	68	0	29	0
	35-59	133	1	41	2
	≥60	92	1	30	1
Diagnosis	Active TB	163	1	44	1
	Uncomfirmed	130	1	56	2
Total		293	2	100	3

### 3.2 Diagnostic accuracy for active TB

**Figure 2:**
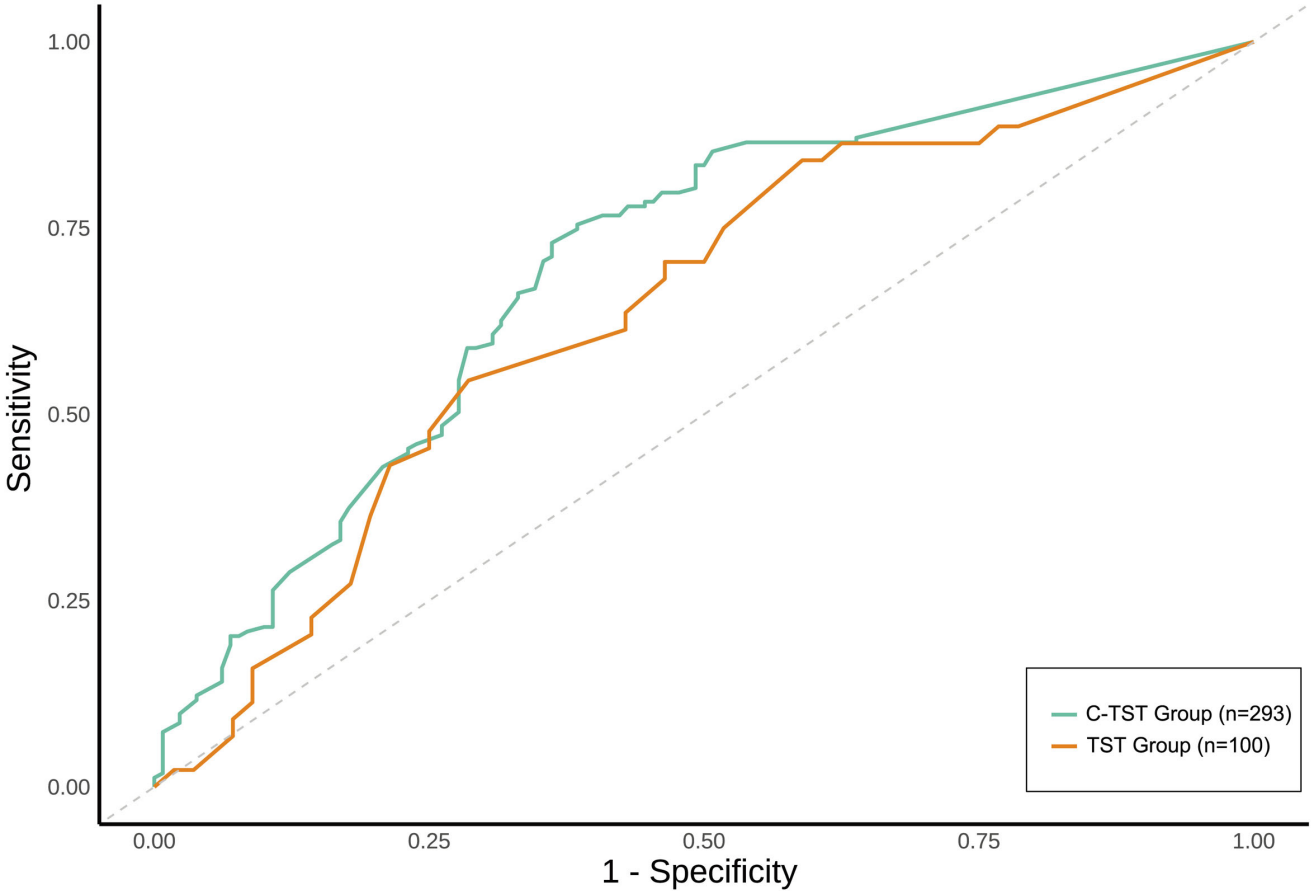
ROC curve for active TB diagnosis in C-TST and TST group. *The ROC curve compares the overall diagnostic efficacy of C-TST and TST for active TB, reflecting the discriminatory ability of the two detection methods.

Compared with the China national diagnostic criteria for active TB, a receiver operating characteristic (ROC) curve was plotted. The Area Under the Curve (AUC) of C-TST was 0.7 (95%CI, 0.64-0.76), and AUC of TST was 0.64 (95%CI, 0.53-0.75). There was no difference in diagnostic efficacy for active TB between the C-TST group and the TST group (*Z*=0.9, *P*=0.37) (**Figure 2**).

In the C-TST and TST groups, the specificity did not reach 70% when the sensitivity exceeded 90%. Both of them did not meet World Health Organization (WHO) target product profile (TPP) targets for TB diagnostics (12).

### 3.3 New features of C-TST

**Figure 3:**
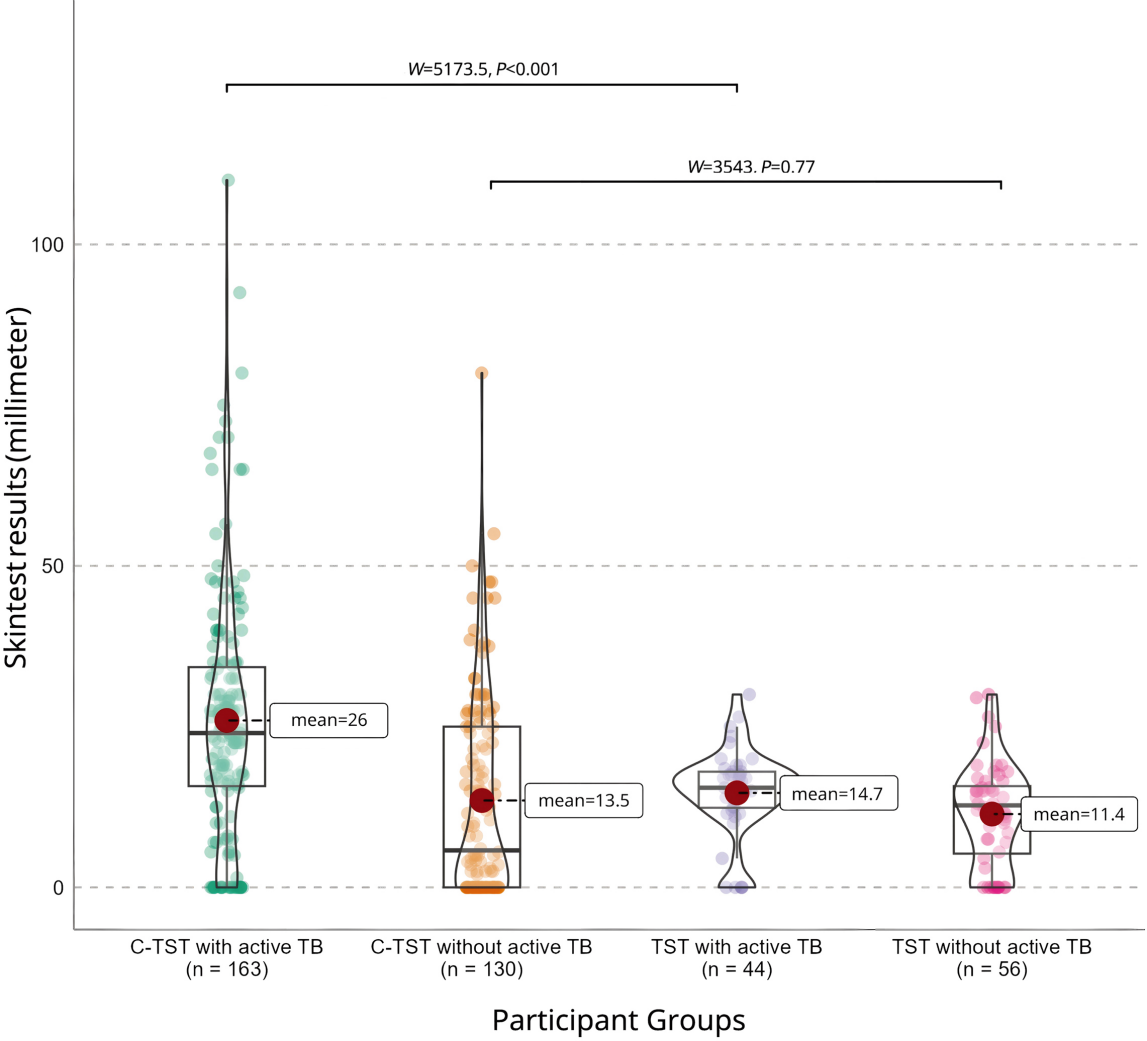
Violin plot and box plot for the distribution of C-TST and TST results. The combined violin and box plot visually presents the median, dispersion and overall distribution difference of diameter between C-TST and TST, which supports the inter-group comparison of immunological response levels.

In terms of diagnostic performance, C-TST has new characteristics compared with TST (**Figure 3**).

In active PTB cases, the median diameter of C-TST induration or erythema was 24 (95%CI, 22.5-27) millimeter, and the median diameter of TST induration was 15.5 (95%CI, 13-17) millimeter. The median diameter of C-TST induration or erythema was significantly larger than that of TST induration (*W*=5173.5, *P*<0.001).

While in unconfirmed participants, the median diameter of C-TST induration or erythema was 5.8 (95%CI, 2.5-13.5) millimeter, and the median diameter of TST induration was 12.8 (95%CI, 9.7-15) millimeter. There was no significant difference between them (*W*=3543, *P=*0.77).

### 3.4 Incremental diagnostic value of C-TST for active TB

When using the third quartile of C-TST with active TB (34.3 millimeter) as the cut-off point, the PPV of C-TST for active TB diagnosis was 74.5% (95%CI, 63.8%-85%). When using the third quartile of TST with active TB (18 millimeter) as the cut-off point, the PPV of TST for active TB diagnosis was 54.5% (95%CI, 35%-73.7%). The PPV of C-TST for active TB diagnosis was significantly larger than that of TST (*Z*=2.46, *P=0.014*).

**Figure 4:**
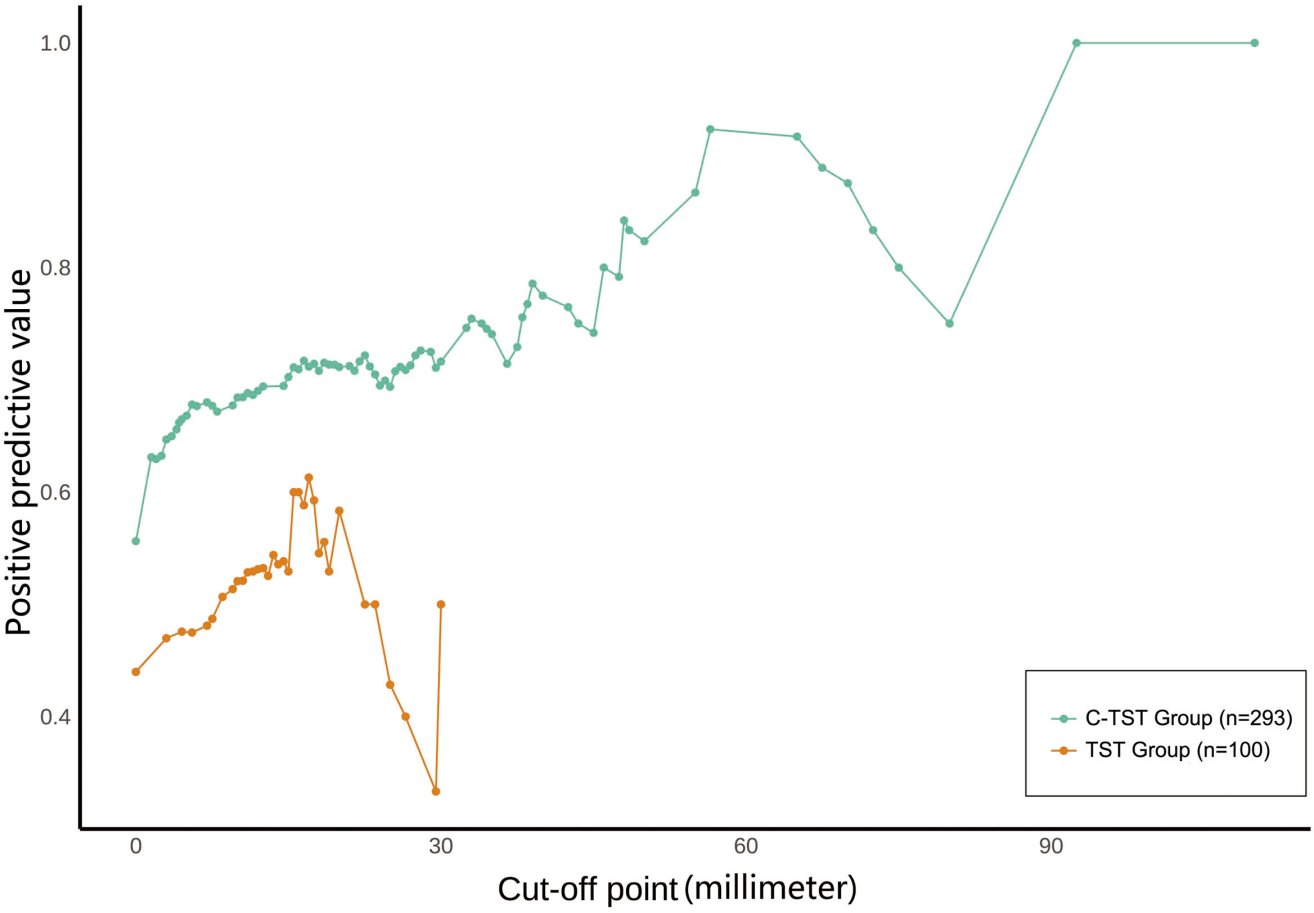
Incremental diagnostic value of C-TST. The curve reflects the changing trend of PPV of C-TST and TST at different cut-off points, and interprets the incremental diagnostic benefit of C-TST compared with TST in clinical screening of active TB.

To further illustrate the diagnostic enrichment of C-TST for active TB diagnosis, the PPV of the C-TST and TST groups at different cut-off points were plotted (**Figure 4**). The PPV curve of C-TST for active TB diagnosis was significantly superior to that of TST (*W*=27940, *P<*0.001).

## 4 Discussion

In the diagnosis of PTB, there has been a continuous effort to discover new diagnostic tools and to explore their potential to enhance the diagnostic efficacy. In this study, the application of C-TST and TST in adult suspected PTB cases showed no significant difference in the overall diagnostic efficacy for active PTB. However, this study has yielded some new findings through the observation of certain characteristics of the C-TST.

C-TST exhibited a significantly larger diameter than TST in active PTB cases, making it easier to identify and acting like a magnifying glass. In the C-TST group, the average diameter in active PTB cases was significantly larger than in undiagnosed participants, and the average diameter of C-TST induration or erythema above the median and 75% quartile showed a lower false-positive rate and a higher PPV for active PTB diagnosis compared to TST, making it possible for more accurate prediction of active PTB. C-TST may have the potential to indicate the presence of active TB above a certain cut-off value in adult suspected PTB cases.

In current research, skin tests such as TBST and TST are primarily used for diagnosing TB infection, rather than for diagnosing active TB. A systematic review found that TBST could offer specific and accurate alternatives to TST and IGRA with similar test performance, which have the potential to improve global tuberculosis control with lower costs and more convenient testing methods (5). It was also demonstrated that the diagnostic performance of the C-TST was close to the T-SPOT.TB assay in the detection of TB infection in the Phase III clinical trial of C-TST (13). In C-TST, an average diameter of ≥5 millimeter is considered the cut-off value for TB infection (14).

Research exploring the clinical value of C-TST in diagnosing active PTB is rare. A study found that C-TST is a suboptimum tool for the differential diagnosis of active TB which is similar to IGRA. However, the kappa value was not high indicating that the cut-off value of C-TST at 5 millimeter may not be appropriate for suspected PTB cases in tertiary specialized hospitals (15). This study did not conduct a quantitative analysis of the C-TST average diameter, and no specific hypotheses were proposed regarding the association between C-TST and the diagnosis of PTB.

A meta-analysis of Diaskintest reporting data from 2009 to 2019 found that the intensity of the immune response of TB cases to Diaskintest may be influenced by TB severity (16).

Not only in C-TST, but also in IGRA, there were some researches on improving active TB diagnostic performance under specific conditions. A prospective cohort study evaluated the diagnostic performance of T-SPOT.TB (T-SPOT) and QuantiFERON-TB Gold (QFT) in detecting active TB in cases with fever of unknown origin in a high TB endemic area by increasing the cut-off values to 125 spot-forming cells (SFCs)/2.5×10^5^ cells for T-SPOT and 4.0 IU/ml for QFT, and the specificity could be improved to >90.0%. The adjusted cut-off values have considerably improved the diagnostic performance (17). A study found that increased cut-off of 173.5 SFCs/2.5×10^5^ for T-SPOT could help diagnose active TB in suspected TB cases in high-burden countries validated in real-world clinical practice (18).

There are a number of reasons for these phenomena. After Mtb infects the body, a series of dynamic changes occur at the levels of gene transcription and protein expression (19). A study found that immune complexes were stimulated by specific antigen ESAT-6/CFP-10 comprising of IgE, IgG3, IgG1, IgG1+IgG3 and IgG1+IgE from IGRA and Diaskintest, and there were significant differences in the level of specific immunoglobulins between active TB and Latent Tuberculosis Infection (LTBI), which may be used as criteria to differentiate between active TB and LTBI (20). The presence of these specific immunoglobulins may lead to a more intense reaction in TBST in active TB.

In C-TST detection, there were still a considerable number of false-negative patients. Diagnosis of TB infection was based on the secretion of IFN-γ in response to Mtb antigens, the absence of which was usually regarded as no infection. A study found that there existed a capability to generate non-IFN-γ immune responses to Mtb in HIV-uninfected healthcare workers who had worked in high TB-exposure environments for 5 years or longer using TST and IGRA (21). This meant that using C-TST could not cover all TB cases.

This study has limitations. This study explored the possibility of improving the diagnostic efficacy of active PTB by increasing the cut-off value of C-TST in areas with a high TB burden. This hypothesis was limited to specific conditions: (1) the subjects were suspected PTB cases, and not applicable to the entire population; (2) the baseline TB prevalence varies across different regions, leading to changes in PPV, and one cut-off value cannot apply a one-size-fits-all approach.

This was a single-center retrospective study, and randomization was not possible in the selection of participants for C-TST and TST. Systematic immunological data were not uniformly collected, immune status could not be adjusted for in the analysis.

C-TST cut-off of 34.3mm was determined *post hoc* rather than pre-specified at study design. Such retrospective threshold derivation may introduce circular reasoning and overestimate PPV. Moreover, this cut-off lacks external validation in independent cohorts, restricting its generalizability.

The sample size was unbalanced between the C-TST group (n=293) and the TST group (n=100). Participant allocation was non-random, based on physician preference and clinical practicality, rather than standardized randomization. Moreover, multivariable adjustment for potential confounding factors was not performed. The intergroup imbalance and absence of confounding control may reduce group comparability and limit causal inference.

Additional multivariable adjustment could not be conducted like bacteriological profile, disease severity and imaging extent, because complete raw data of several critical confounding variables cannot be fully retrieved. A larger induration size may reflect overall disease burden rather than diagnostic specificity alone.

This study lacked a blinded design for skin test interpretation. Since diagnostic work-up results were often available before skin test reading, complete blinding was not feasible, which may introduce inevitable observer bias.

Accordingly, our diagnostic performance analysis should be interpreted as exploratory, not confirmatory. Further prospective randomized controlled trials and external validation adopting independent blinded reading are required to confirm the clinical utility of C-TST.

## 5 Conclusion

In conclusion, immunological tests based on ESAT-6 and CFP-10 antigens, such as C-TST, are primarily used for diagnosing TB infection. But in clinical practice, under specific conditions, they may have the potential to further assist in the diagnosis of active PTB, and it needs further research.

## Data Availability

The TB infection tests data of this study has been stored in the provincial TB information system but restrictions apply to the availability of these data, which were used under license for the current study, and so are not publicly available. Data are however available from the corresponding author upon reasonable request and with permission of the provincial TB information system.
